# An economic assessment of alternative antimicrobial use scenarios on pig farms

**DOI:** 10.3389/fvets.2024.1381499

**Published:** 2024-04-30

**Authors:** Shailesh Shrestha, Maria R. da Costa, Carla Correia-Gomes, Amanda Nevel, Andy McGowan, Sue C. Tongue

**Affiliations:** ^1^Central Faculty, Department of Rural Economy, Environment, and Society, SRUC (Scotland's Rural College), Edinburgh, United Kingdom; ^2^Centre for Epidemiology and Planetary Health, School of Veterinary Medicine, SRUC (Scotland's Rural College), Edinburgh, United Kingdom; ^3^Animal Health Ireland, Leitrim, Ireland; ^4^Agriculture and Horticulture Development Board (AHDB) Pork, Coventry, United Kingdom; ^5^Wholesome Pigs (Scotland) Ltd., Aberdeenshire, United Kingdom

**Keywords:** pig farm, economic assessment, antimicrobial resistance, antimicrobial usage, farm level modeling

## Abstract

This paper explores the theoretical economic outcome of management changes that result in different levels of antimicrobial use (AMU) in two types of UK pig farm. A static farm economic pig production model (FEPM) was used on a representative ‘Top-third’ most profitable farm and a representative ‘Mid-range’ profitable farm. Three AMU theoretical management scenarios were investigated; (a) management changes leading to a reduction of AMU by 35% (AMU35); (b) more extensive management changes leading to a reduction of AMU by 95% (AMU95); and (c) implementing depopulation of the herd (AMU Depop). A sensitivity analysis was conducted to determine the effect of increases or decreases in pig revenue and feed price on farm gross margin under these scenarios. Over a single year, the AMU35 scenario was estimated to have a small positive impact (+3%) on both farm types. The other two AMU reduction scenarios had higher AMU reduction on farms but required higher variable cost and hence they resulted in lower farm profitability. There was a substantial reduction (up to −50%) in farm gross margin under these two AMU reduction scenarios in the modeled short-term time-period. The impact of the alternative AMU scenarios was slightly higher on a farm representing the ‘Top-third’ farm type, reducing farm gross margin further by 7% compared to the ‘Mid-range’ farm. Nevertheless, both farm types stay profitable under all three AMU scenarios. The results showed that in the modeled short-term implementing management changes that result in a reduction of on-farm AMU by 35% had a good economic outcome. In practice, the other two scenarios would be considered as longer-term strategies. Although both require higher initial costs to implement, the improved biosecurity and hygiene will benefit from lower disease occurrence for a longer term. Farm gross margins were, however, found to be highly sensitive to changes on market prices especially increasing feed prices. An increase of more than 15% in feed price moved a profitable farm into a loss-making farm. It will be economically challenging for uptakes of these, or similar, AMU reduction scenarios on farms if the market prices become un-favorable to pig farmers.

## 1 Introduction

Antimicrobial resistance (AMR) is a steadily increasing threat to public and livestock health around the world (1). Since the introduction in the 20th century of antimicrobial drugs (such as antibiotics) to treat infections, the rate of emergence and spread of AMR has increased substantially (2, 3). This has led to a situation where many common bacterial infections are, once again, becoming difficult to control. AMR is a multi-factorial problem that will require multi-sectorial action to address. Reduction of over-reliance on and inappropriate use of antimicrobials has been outlined by leading health researchers and agencies as the main strategy available to tackle the challenge of increasing AMR globally. This applies to all sectors; for instance, the WHO Global Action Plan stated a need ‘*to optimise the use of antimicrobial medicines in human and animal health*’ (4) and a ‘One Health’ approach, combining human, animal and environment aspects has been advocated (5) as ‘*the main strategy required to reduce the development of antimicrobial resistance*’.

The potential risk to human health from the use of antimicrobials in food animals has been highlighted by many researchers (6–16). More recent evidence is suggesting that while there is still a risk of spread between species, including humans, most AMR is and remains species specific (17, 18). Nevertheless, it is incumbent on all sectors to use antibiotics responsibly to preserve their efficacy for as long as possible. In addition to therapeutic use and metaphylactic (i.e., administration to clinically healthy animals that belong to a herd or flock with clinical signs) antimicrobial substances have been, or are being, used in livestock in some parts of the world not only for routine prophylaxis (treatment of animals at risk of infection in the absence of clinical signs) but also for enhancement of production (e.g., growth promoters); in other areas these latter two uses have been banned, or restricted. Responsible use is about as little as possible but as much as necessary (to protect animal welfare). While AMR is a global challenge, the degree of control and regulation of antimicrobial use varies widely despite some practices being major contributory factors to the development of AMR in livestock (19–21). The economics of production within a sector may contribute to the ability to adopt and to implement policies to reduce AMU.

Pork meat is one of the major sources of human food with over 40% of total meat consumed worldwide coming from pigs (22). As in other livestock sectors (1, 23, 24), the use of antibiotics (the main antimicrobial agent) has been a common practice on pig farms. The pig and poultry sectors are often considered to use more antimicrobials compared with other food animal production systems. An effort to estimate global consumption of antimicrobial in food animals suggests that the pig sector uses almost four times more antimicrobial per kilogramme meat produced (25). In the United Kingdom (UK), a quarter of all the antimicrobials sold in 2017 were used in farm animals (26). Around 36% of the total antimicrobials sold in 2022 for animal use were bought by pig farmers (27). The reported frequency of evidence for AMR from pigs and pork products varies. For instance, a study in China showed 91% of *E. coli* isolates from 1871 samples from pigs and their breeding environment contained *E. coli* resistant to major antimicrobials (22). In the UK, an abundance of AMR genes has been demonstrated in a single herd (28). At a population-level, it is reported (27) that there has been an encouraging increase in fully susceptible *E. coli*. There has been a concomitant increase in the percentage of pigs carrying ESBL/AmpC^1^ - producing *E. coli* bacteria (27). This increase is not accompanied by an increase in clinical cases of resistance (Nevel pers. comm).

Antibiotic stewardship programmes, co-ordinating efforts to reduce antimicrobial use and encourage responsible and prudent use in the UK livestock sectors have been developed and implemented (29). This should help to preserve the efficacy of antibiotics for as long as possible. These activities have been especially effective in the pig sector. They are proving worthwhile with the UK pig sector having achieved, in 2022, its aim to reduce antimicrobial use (AMU) by 30% compared to the year 2020 levels (29, 30). This aim was initially set to be achieved by the year 2024. Indeed, since antimicrobial use in the pig sector started being recorded in 2015, the sector has reduced its use by 75% as of 2022 (30).

Pig producers will need to continue their efforts to use antibiotics responsibly thereby protect the efficacy of currently available products and protecting the reputation of the sector. Responsible antibiotic use is an important issue for trade. Actions will be needed at both industry-wide and individual farm level. There are several studies of alternative management options that can be used to reduce antimicrobial use on livestock farms (2, 31). To reduce antimicrobial usage without adversely affecting farm production and farm profitability is a challenge and it is necessary to make (holistic) changes, such as improvements in facilities, management, and health status. To replace antimicrobials, farmers need to minimize production losses by adopting strategies aimed at preventing disease occurrence on farms. These strategies could include adopting vaccination programmes, adding supplements to feed to boost immune systems and changing farm management to improve hygiene and biosecurity on farms (31–39) (40). A list of alternative strategies to reduce antimicrobial use on farms, as extracted from the studies cited, are:

- improved biosecurity on farms;
- change in management such as adopting ‘all-in-all-out’ system, reduced stocking density;
- use of vaccines;
- improved diagnostics to improve appropriate drug selection and dosage;
- improved hygiene on and around farms including waste management;
- use of feed additives such as enzymes, metal (e.g., copper and zinc), organic acids, essential oils and probiotics;
- the use of genetically improved, or modified to be less susceptible to disease, stock;
- increased in-water dosing instead of in-feed treatment.

These management options need to be economically and practically feasible to be successfully implemented on farms. There are many economic studies that analyze the decision making on farms due to certain changes in policies or markets (41–44). Generally, the economic consequences of implementing alternative management strategies on farms are one of the major barriers for farmers to change their farm management strategies (45–47). There are few studies published that investigate the economics of AMU and AMR in livestock species (48, 49, 50, 51) and specifically on pig farms (39). In this manuscript, we describe the use of a farm level economic pig production model (FEPM) to assess the economic impacts on profitability in the immediate term, of the adoption of some common options included in three scenarios. A sensitivity analysis illustrates, for the first time, the relationship between the impacts of these scenarios and other factors that influence the profitability of UK pig farms.

## 2 Materials and methods

In this study, a static, farm level, economic pig production model (FEPM) was used to explore the economic effects of the adoption of alternative antibiotic use management options on two farms with different profitability levels over a calendar year. Farm gross marign was used as a measure to compare the economic impact of alternative management options with a baseline scenario. A sensitivity analysis was included in the model to examine the effect of price changes on the modeling outputs.

### 2.1 Farm data inputs

Farm data for representative pig farms from two different theoretical indoor farm types were used to capture potential differences in the impact of changes due to different farm structure and management. The two farm types were distinguished from each other by their level of profitability (farm gross margin). The input data were for firstly an average farm representing the ‘Top-third’ most profitable producers (known hereafter as the Top third farm) and secondly an average farm representing the ‘Mid-range’ of the remaining two-thirds of pig farms in the UK (known hereafter as the ‘Mid-range’ farm). The farm level data used for these two farming systems (Table 1) was taken from several sources including AHDB (52), Farm Management Handbook (53) and QMS Agrosoft (54). Most of the physical parameters such as sow numbers, family labor available and labor price were kept similar in both farm types to facilitate comparison of the outputs between the two. The management difference between these two farm types is presented by differences in finisher revenue, replacement rate, litter size and litter rate. These differences highlight a difference in production efficiency between these two farm types, which results in their different status regarding profitability.

**Table 1:** Farm variables and parameters for a ‘Top third’ farm and a ‘Mid-range’ farm.

Farm parameter	Farm type	
	‘Top third’ farm	‘Mid-range’ farm
Sow numbers	70	70
Family labor (MU)*	2.5	2.5
Replacement costs (£/animal)	205	205
Culled price (£/animal)	120	120
Weaner revenue (£/animal)	55	55
Finisher revenue (£/animal)	139.8	132.7
Labor price (£/hr)	8.91	8.91
Replacement rate (%)	61	55
Sow mortality (%)	7	6
Litters per sow per year	2.5	2.3
Piglet (pre-weaning) mortality (%)	10	10
Weaner/finisher mortality (5)	5	5
Litter size	14.2	13.3

*MU = man unit (1 MU = 2,200 h/yr).

Farm parameters required by animals in different age and production categories were taken from the Farm Management Handbook (53) and are listed in Table 2.

**Table 2:** Additional farm parameters used for each pig categories.

Parameters	Farm type	Pig category				
		Sow	Sow (farrowing)	Piglet	Weaner	Finisher
Feed requirements (kh/yr)	‘Top third’	650	650	86	346	1,008
	‘Mid-range’	650	650	75	299	873
Variable costs (£/yr)	‘Top third’	87	87	0	0	6
	‘Mid-range’	85	85	0	0	6
Feed costs (£.kg)		0.23	0.23	0.8	0.5	0.25
Labor requirements (hr/yr)		14	14	0.2	2.3	2.3

#### 2.1.1 Farm level economic pig production model (FEPM)

The static, farm level economic pig production model, FEPM, is based on ScotFarm, a farm level economic model that was developed at Scotland’s Rural College, SRUC (55). ScotFarm has been used in several studies to conduct impact assessment of policy and management changes on Scottish dairy, beef, sheep and arable farms (56–60). The livestock system used in ScotFarm is modified into pig production system to develop FEPM. The FEPM is a linear programming (LP) model and maximises farm gross margin subject to available farm resources (such as labor) and management over a production year. The model is based on farming system analysis technique which includes biophysical and management relationships interlinking production to farm resources. For example, the model links availability of labor (both family and hired labor) and feed to individual animal requirements and projects the number of animals in five different age categories that can be kept within a production cycle. The model determines farm gross margin as total revenue generated from sold animals minus feed costs, labor costs, management costs and antimicrobial costs as shown in equation (1). The management costs include costs of interventions to improve hygiene and biosecurity such as installation of foot bath, ventilation, shower and fences. The antimicrobial costs include cost of antibiotics used in feed and water for the animals.


(1) ρ=rev∗pig−fp∗fq−lp∗l−mc−amc∀farmtypes

Where, ρ = farm gross margin, rev = pig revenue, pig = number of pigs sold, fp = feed price, fq = quantity of feed used, lp = labor price per hour, l = total number of labor hours used, mc = management costs, amc = antimicrobial costs.

**Figure 1:**
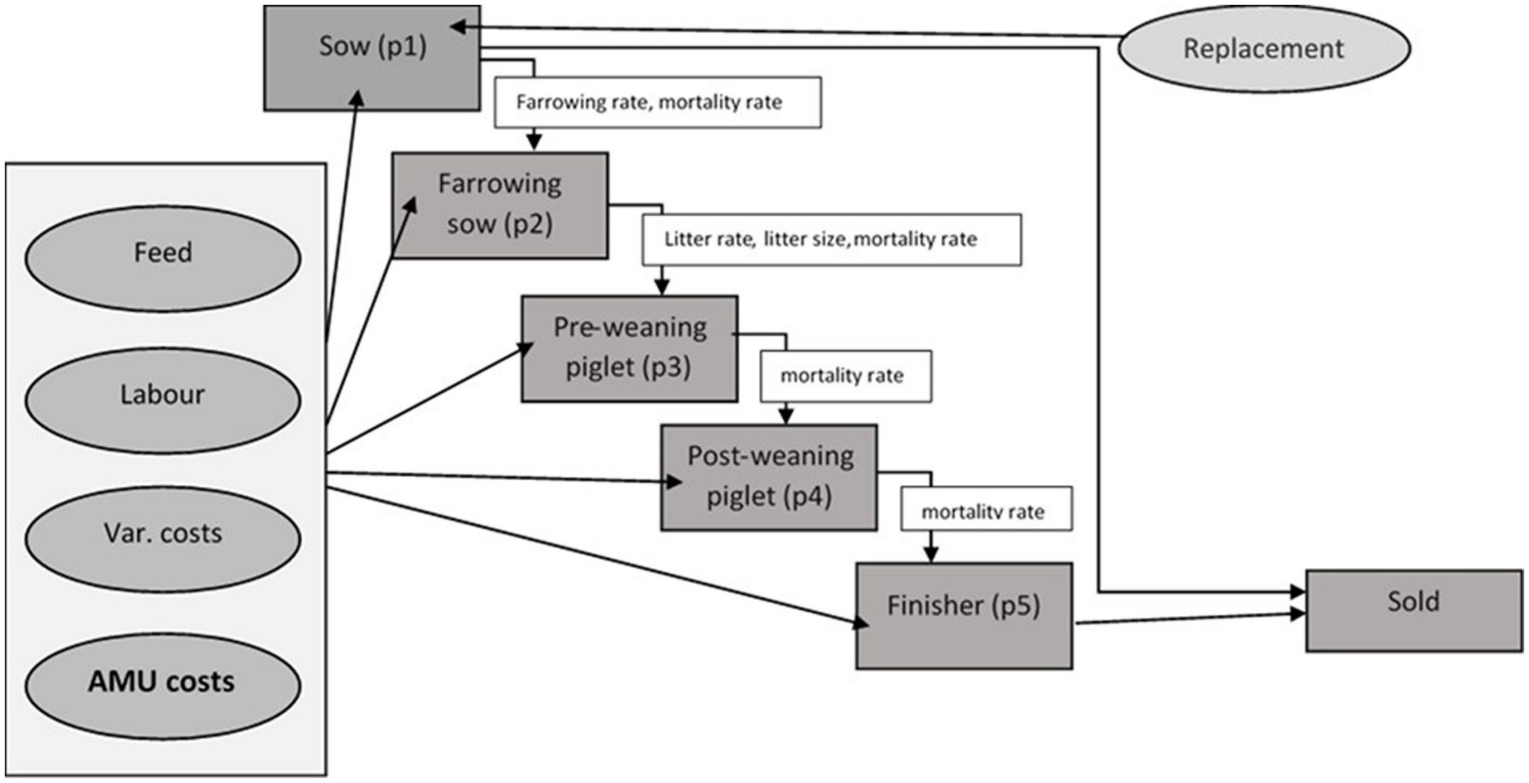
A schematic diagram of the FEPM.

The model is parametrised to represent existing management practice on two representative pig farm types. The model is based on a typical farrow-to-finish (or “birth to bacon”) pig farm. It uses five age groups (*pt1* = sow, *pt2* = farrowing sow, *pt3* = pre-weaning piglets, *pt4* = post weaning piglets and *pt5* = finishers). These age groups are linked to each other and to the available farm resources such as feed and labor (Figure 1). All the animals in *pt5* age category (finishers) are sold and farm revenue is thus generated.

The pigs progress from one age group to another over time within a production cycle following constraining coefficients such as farrowing rate (*α*), mortality rate (*μ*) as shown in equation (2) below as an example to determine pre-weaning pigs (*pt3*).


(2) $$pt3=pt2*\alpha *\mu_{pt3}$$

*Where, t = 1,2,3,4,and 5.*

Each of the pig age group is constraint over feed and labor requirement. The coefficients for feed (*frt*) and labor (*lrt*) requirement are based on published figures (52, 54) and an Irish pig production model (61). Total quantity of feed used (*fq*) per production cycle is determined as shown in equation (3).


(3) fq=pt∗frt

*Where, t = 1,2,3,4,and 5; fr = feed requirement coefficient.*

Determination of total labor used (*l*) per production cycle followed a similar process using the labor requirement coefficients (*LRt*) for each age group.

The FEPM is run for only one production year to capture short-term decision making on farms. The presence of a highly volatile pig meat and feed market (39, 62–64) and a very dynamic pig health status on farms (65, 66) make it difficult to model pig farmers’ long-term decisions. Although, this paper focuses on short-term decision making, a sensitivity analysis of pig market price is conducted, nevertheless, to examine the impact of volatility in pig market price on farmers’ decision making.

#### 2.1.2 Antibiotic reduction management options

Three feasible alternative management options were selected based on discussion with industry experts. These discussions included pig farm/production experts, veterinarians, farm consultants and semi-structured discussions with a selection of pig farmers from across the UK (67). They, however, are theoretical.

The three alternative management scenarios selected for this study were: (i) improving farm hygiene leading to a reduction in antibiotic use on farms by 35%; (ii) improving farm biosecurity, hygiene leading to a reduction in antimicrobial use on farms by 95% and (iii) improving biosecurity, hygiene, leading to a reduction in antimicrobial use by 95% and adopting a true ‘all-in-all-out’ management strategy. These options were considered to be the most likely management options that could be adopted by pig farmers in the UK for the following reasons: option (i) the most easily implemented option that was considered feasible by the experts consulted, with minimal investments on a farm; option (ii) is an hypothetical but relatively easy to implement scenario with a higher level of investment and risk, in which 95% (by volume) of in-feed antimicrobials are removed; option (iii) was seen as the most likely solution to effectively remove 95% of in-feed antimicrobials without impairing the health and welfare of animals. In this option, the herd is depopulated, and the farm is then repopulated with healthy animals. This would ensure the highest health status and the implementation of appropriate management and biosecurity changes would perpetuate that status.

#### 2.1.3 Modeling scenarios

The model runs on a ‘baseline’ scenario with three alternative management scenarios.

The farm gross margin estimated under the ‘***alternative AMU management***’ scenarios are compared with the farm gross margin under the ‘***baseline***’ scenario to determine the economic impacts of those alternative managements.

##### 2.1.3.1 The baseline scenario

This scenario represents the existing management conditions on an average pig farm of the specified farm type. This scenario was used as a comparison point against which the impacts of each of the three alternative management scenarios were measured.

##### 2.1.3.2 Alternative AMU management scenarios

The alternative management scenarios are the three different AMU reduction scenarios that can be implemented on a farm. The assumptions made for these scenarios are as follows (Table 3):

- **AMU35 scenario**: This scenario restricts the use of antibiotics in feed on farm to 65% of that in the baseline scenario (i.e., a 35% reduction in-feed AMU). It is assumed that farm variable costs increase by 10% to improve sanitation and hygiene as well as animal welfare on farm. This scenario assumes that improved hygiene, sanitation, and welfare of animals will increase farm production by 10%.
- **AMU95 scenario**: This scenario entirely removes the use of antibiotics in feed on a farm. It is assumed that this amounts to 95% reduction of in-feed AMU in volume. The scenario assumes that farms will improve biosecurity to minimize disease and infections. The farm variable costs are, therefore, doubled to accommodate some additional management changes such as increasing hygiene practices, cleanliness, increase use of disinfectants, control vermin etc. As under the **AMU35 scenario**, it is assumed that the productivity will improve by 10% under this scenario.
- **AMU Depop scenario**: Under this scenario, the entire pig population is removed from the shed for a certain period. The number of litters per sow is reduced to only one per year and the entire herd is replaced after a production cycle. The AMU is reduced by 95% in-feed use and farm variable costs are increased by 50% to accommodate activities to improve sanitation and hygiene on the farm. Farm production is assumed to increase by 10% as under other two AMU scenarios.

**Table 3:** Model scenarios and their assumptions.

	Baseline	Alternative AMU management scenarios		
		AMU35	AMU95	AMU De-pop
AMU reduction	0%	35%	95%	95%
Production level	100%	110%	110%	110%
Variable costs	100%	110%	200%	150%
Replacement rate*	61%/55%	61%/55%	61%/55%	100%
Litter per sow*	2.5/2.3	2.5/2.3	2.5/2.3	1%

*for the two farming systems (‘Top third’/‘Mid-range’).

A general assumption that an improvement in hygiene and biosecurity will improve overall production level of the production system was considered under these alternative AMU scenarios. This assumption of 10% improvement in productivity is based on findings of improved production under improved management practices (31, 32, 39, 61, 68).

**Figure 2:**
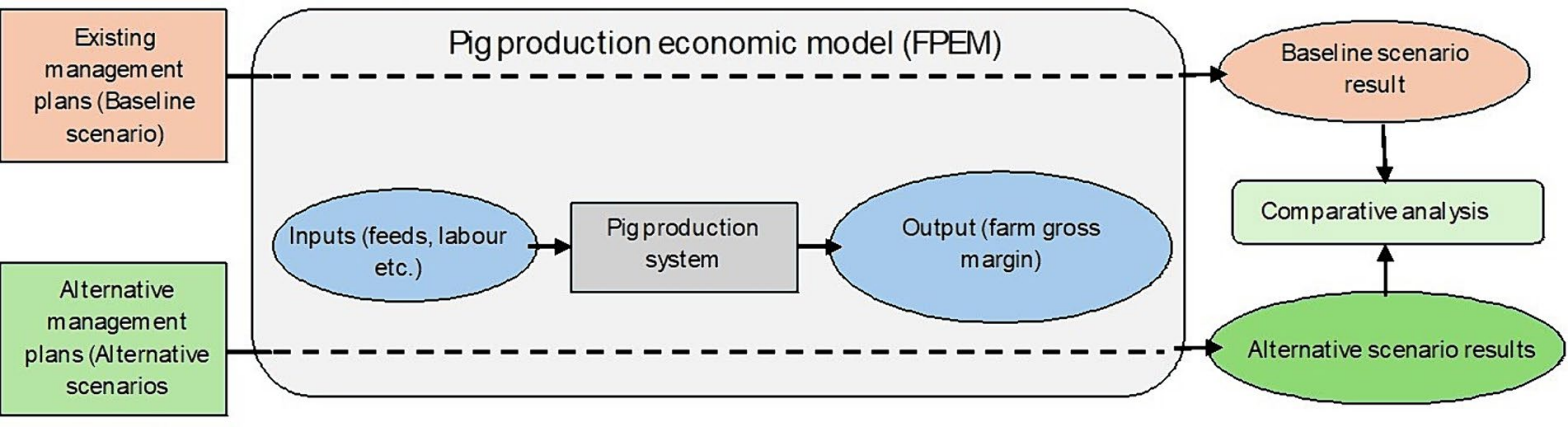
The modelling workflow.

As described above, the ‘baseline’ scenario presents a status quo scenario for both pig farm types. The three ‘alternative AMU management’ scenarios are different from the ‘baseline’ scenario in regard to the AMU and changes in costs associated with improvement in the hygiene and biosecurity status of the farms. Farm gross margins from the three alternative AMU management scenarios are compared with the farm gross margin under the baseline scenario to determine the impact of alternative AMU managements used on farms under those three alternative scenarios. A schematic diagram to represent this workflow is presented in Figure 2.

#### 2.1.4 Sensitivity analysis

**Figure 3:**
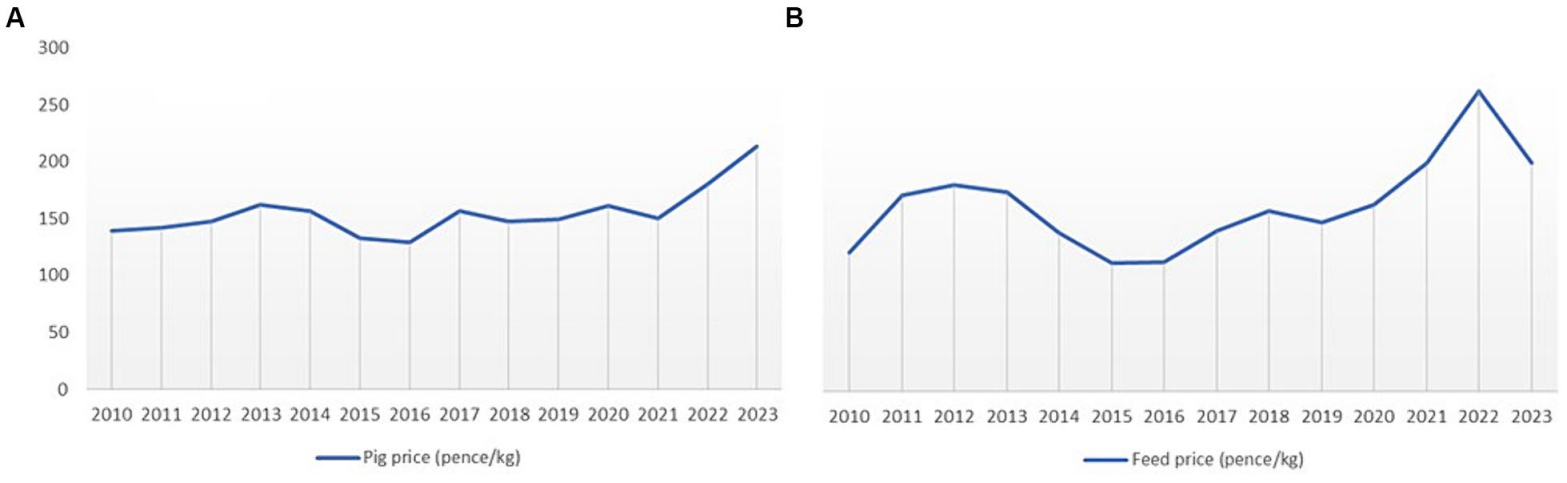
Historical changes in **(A)** standard pig price and **(B)** feed price over the last 14 years.

A sensitivity analysis was conducted to examine the effects of changes in pig revenues and feed price on farm gross margin under the alternative AMU management scenarios. Feed price constitutes around 60–80% of total cost of production in major pork producing countries worldwide (69). These price parameters were chosen for the sensitivity analysis because the UK pig market price and feed price have fluctuated substantially over the past decade (Figure 3). The large variation in these prices creates uncertainty when estimating the impacts of future management changes on farm margins. To examine this uncertainty, a sensitivity analysis with changes (±5, ±10 and ± 15%) on pig revenue and feed prices was conducted to determine the influence of those variabilities on farm profit under the alternative AMU management scenarios used for this study. The parameters for the sensitivity analysis were derived from the most frequent pig price changes in the UK market in the last 14 years.

## 3 Results

### 3.1 Farm gross margin

**Figure 4:**
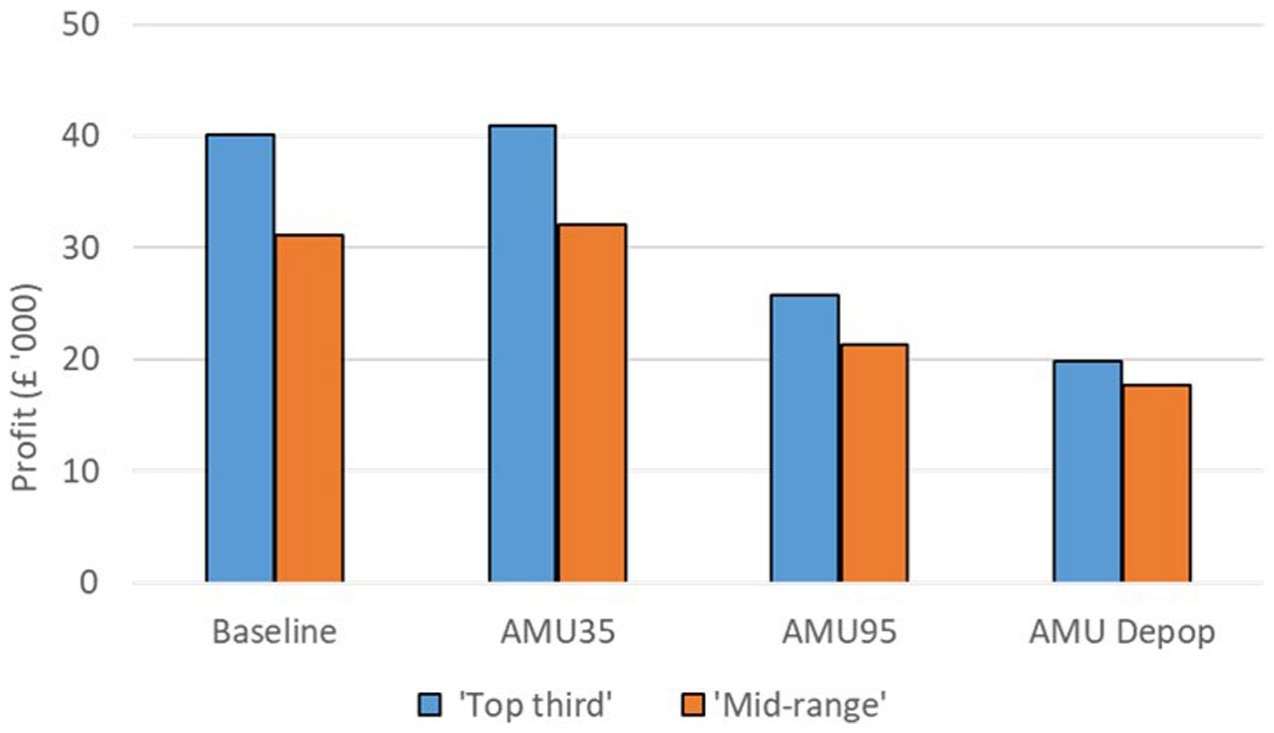
Farm gross margin under the baseline and three alternative AMU management scenarios for a ‘Top third’ and a ‘Mid-range’ farrow-to-finish farming system.

The farm gross margin for a farrow-to-finish pig farm in the ‘Top third’ of profitability group is estimated to be £40,103 whereas and that for a ‘Mid-range’ pig farm is estimated to be £31,172 in the baseline scenario. The impacts of the alternative AMU management scenarios on each of these two farm types are shown in Figure 4. For both farm types, there is a slight but positive increase in farm gross margin (2% increase in the ‘Top third’ farm and 3% increase in the ‘Mid-range’ farm) when AMU is reduced by 35% under the **‘AMU35’** scenario. The reduction in AMU costs as well as the increased production has led to this positive change despite the increased variable costs.

There is a large reduction in farm gross margin when AMU is reduced on farms by 95% under the ‘**AMU95**’ scenario. This occurs because the large increase in variable costs results in a substantial decrease in farm gross margin, with a 36 and 32% reduction in farm gross margin on both ‘Top third’ and ‘Mid-range’ farms, respectively. Under this scenario the assumed increase in production does not compensate for the increase in variable costs.

The ‘**AMU Depop’** scenario also led to substantial reduction in farm gross margin for both farm types with 50% reduction on a ‘Top third’ farm and a 43% reduction on an average pig farm. A substantial increase in variable costs coupled with higher replacement costs were the reason for this large drop in farm gross margin under this scenario.

### 3.2 Sensitivity analysis

**Figure 5:**
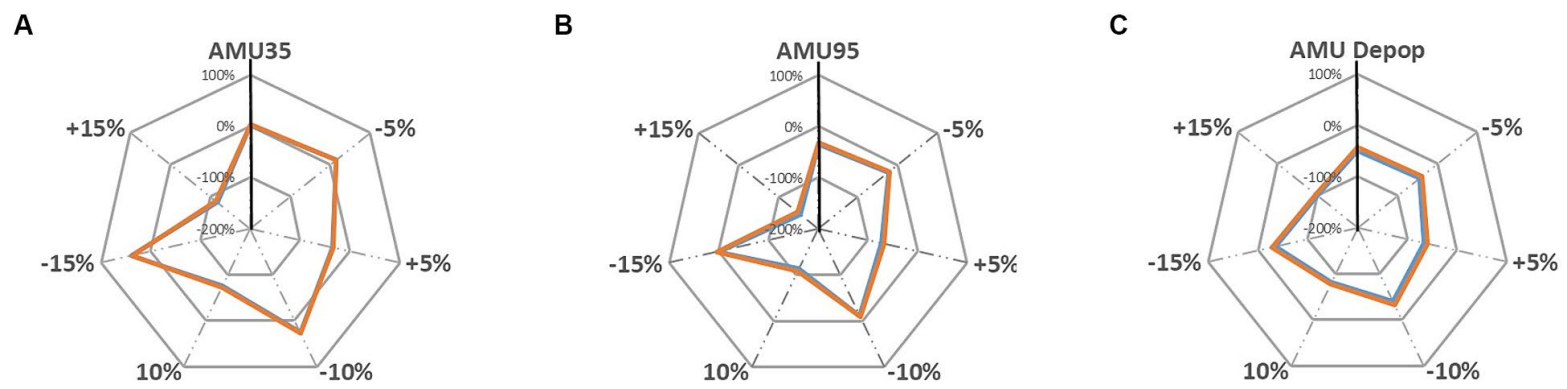
Sensitivity analysis of changing pig revenue ±5%, ±10 and ± 15% on farm gross margin under; **(A)** AMU35, **(B)** AMU95, and **(C)** AMU De-pop scenarios [‘Top third’ farm, ‘Mid-range’ farm].

**Figure 6:**
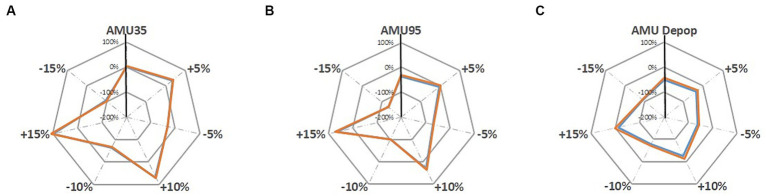
Sensitivity analysis of changing feed price (±5%, ±10 and ± 15%) on farm gross margin under; **(A)** AMU35, **(B)** AMU95, and **(C)** AMU De-pop scenarios [‘Top third’ farm, ‘Mid-range’ farm].

Under the sensitivity analysis the farm gross margins are further changed under each sensitivity analysis scenarios which are illustrated in Figures 5, 6. The grey dotted lines in these two figures represent the sensitivity analysis scenarios where the pig revenue/feed price are changed to ±5, ±10 and ± 15%. The grey solid line shows the percentage change (for instance, 100, 0, −100% and − 200%) compared to the baseline. The solid vertical black line represents the changes in farm gross margin under three alternative AMU scenarios that are presented in Table 4. The colored lines are the farm gross margins on the ‘Top third’ farm (orange line) and on the ‘Mid-range’ farm (blue line).

**Table 4:** Changes in farm gross margins for the two farm types under three alternative AMU management scenarios compared to the baseline scenario.

Farm type	AMU 35%	AMU 95%	AMU De-pop
Top third	2%	−36%	−50%
Mid-range	3%	−32%	−43%

The results suggest that farm gross margins on both pig farm types are highly sensitive to changes in pig revenues and there was only a very slight difference in impact on margins between both farm types, hence, the red and blue lines in Figures 5, 6 are overlapping and difficult to distinguish from each other. When pig revenue increased, farm gross margin increased for all three alternative AMU management scenarios as expected (Figure 5). Farm gross margin decreased substantially when the revenue decreased by 15%. The margins decreased substantially by up to 96% when revenues reduced by 15% under the AMU35 and AMU De-pop scenarios. Farms under the other two AMU management scenario, nevertheless, still break even at this reduction in pig revenue and start making loss only beyond a 15% reduction to pig price. However, under AMU95% scenario, 15% decrease in farm revenue projected the farm gross margin to move beyond 100% suggesting the farms will move from being profitable to a loss-making farm.

Changes in feed price show a larger sensitivity on farm gross margin compared to the changes in pig revenues (Figure 6). Reduction in feed prices have positive impact on farm margins on all three alternative scenarios. Farms move from being profitable to making a loss when feed price increased by 15% under all three scenarios. Under the AMU95 scenario farm start making a loss at an increase of 10% to feed price suggesting that this scenario is more sensitive to increase in feed price than other two alternative AMU scenarios.

## 4 Discussion and conclusion

Farmers make decisions on management strategies to achieve farm targets, such as to expand production, to increase profitability, to reduce greenhouse gas (GHG) emissions, or as assumed in this paper, to reduce antimicrobial use on farms. One of the main drivers influencing farm level decisions on different production systems is the economic aspect of those decisions (70–72). Economics become much more important in decision making on commercial farms like poultry, pig and dairy farms (39, 59, 73–75). Understanding the economic feasibility of farm level decision making provides essential insight into the pros and cons of management strategies on farms. This paper looked at the economic feasibility of different AMU reduction scenarios using a static economic pig production model (FEPM). The modeled period was 1 year. The FEPM is a modified version of ScotFarm, a farm level economic model developed at SRUC to conduct impact assessment of external shocks to an agricultural production system. The ScotFarm model has previously been used to assess economic impacts of policies (57, 60, 76), to explore management scenarios reducing GHG emissions (56, 77), and to determine the financial burdens of livestock diseases on farms (58, 59). The FEPM model was run on two types of pig production systems in the UK. These farm types were selected on the assumption that economic impacts of management changes would be different for different farm types (72, 78). Matheson et al. (79) also found that the reduction in amount of antimicrobial use between 2017 and 2018 was different between pig farms in the UK based on their characterisation.

This type of modeling study does not and should not be used to provide direct advice for individual farms; it explores generically the comparative effects of adopting different scenarios in a simplified, representative, system. The three alternative AMU reduction scenarios used in this study were identified by the pig experts as the most plausible scenarios for implementation on pig farms in the UK. These three scenarios of implementation are relatively common across the industry, though it is important to note that not all scenarios are equally applicable – individual farms need to decide whether any of these scenarios are feasible and suitable within their context. For example, depopulation option as considered under the ‘AMU Depop’ scenario may not be adequate to prevent a higher risk of reinfection if there are neighboring farms which are not adopting similar measures to prevent disease on farms.

The economic and production parameters under these scenarios were based on published literature and were checked with experts to be appropriate, (i.e., valid), values. The parameters used in the model are very similar to the parameters used for Irish pig production (61). It was assumed that production would increase by 10% due to improved hygiene and biosecurity in all three scenarios. It can be argued that some farms may have more than 10% increase in production level due efficient and improved management conditions on farms, and due to the overall higher health status, particularly in the AMU Depop scenario. Indeed, Sasaki et al. (68), documented the effect of depopulating and restocking on three farrow-to-finish Japanese pig farms on reproductive and production performance. These authors used 2 years’ worth of data before the depop and 2 years’ worth of data after the repop. They found that postweaning mortality and age at slaughter (days) reduced by 58 and 11% respectively, while average daily gain (g) increased by 12%. However, we kept the 10% improvement assumption in all scenarios for this paper because, firstly, this assumption was based on studies for pig farms undertaken on European pig production system that are similar to the UK pig farms used in this paper and secondly, we did not find any other peer reviewed paper apart from the Sasaki paper to suggest a higher increase in productivity.

This study showed the monetary benefits of making management improvements while reducing AMU by 35% on farm. The other two AMU reduction scenarios appear to have led to decreased farm gross margin. However, there are a couple of important considerations that must be made to understand these results. First, we used a static model for this work, meaning that all costs were incurred in one time point and the returns must be seen in the context that the time frame for the profitability estimate is one calendar year. The two less-profitable scenarios required major investments, such as improvements in management and biosecurity, or the depopulation and repopulation of the farm. The economic return of such interventions is likely to be seen over time and to increase with each additional production cycle run after the changes were implemented. For instance, a Dutch study showed a reduction of around £50 in animal health costs per sow over the years by implementing de-populationmanagement system (80). Second, we assumed that the farm’s health status is stabilized with the improved management and specific pathogen free claims and the likelihood of disease outbreaks is reduced substantially over the years. In the field, for individual farms this may not be the case, for instance, the benefits of depop-repop can be lost immediately if disease recurs through a breach in biosecurity.

The results also showed that reducing AMU under the described scenarios would have a slightly higher adverse impact on a ‘Top third’ farm compared to a ‘Mid-range’ average farm. A ‘Top third’ farm has, in general, higher input costs compared to an average ‘Mid-range’ farm. Farms with higher input costs are known to be more vulnerable to increased variable costs compared to farms with lower input costs (81).

This study highlighted the impacts of changes in pig revenues and feed costs. The high volatility of these costs and prices make decision making on farms very difficult. There has been an increase in average pig market price in 2023 by almost 38% compared to 2018 pig prices in the UK (62). However, feed costs also increased by a substantial 78% due to higher grain prices (62). Although, these changes in market prices are substantially higher than the changes used in the sensitivity analysis (±5 to ±15%) in this study, the results show that an un-favorable pig price and feed costs would make famers’ adoption of these AMU management scenarios financially challenging. These aspects need to be considered seriously when developing AMU reduction strategies and policies. Due to recent high uncertainty in market prices, there have been recommendations to reduce feed costs on pig farms by increasing feed conversion efficiency, reducing slaughter weight and the use of alternative diets (82). These recommendations are however beyond the scope of this study and hence were not included in the model.

In conclusion, it seems to be possible to reduce AMU while still maintaining a profitable farm, as long as the AMU reduction is done in conjunction with farm and management improvements. Under the assumptions of this model, a short term decision making management changes coupled with a small AMU reduction on farm is attainable and translates into higher margins over 1 year. Although our model predicted that more drastic AMU reductions incurred a loss of farm gross margin compared to the baseline model, it is likely that over time the economic return will be favourable and translates into higher margins. Market fluctuations and feed prices are major factors affecting pig production and the profitability of pig enterprises currently; they must be considered when making decisions about antimicrobial use reduction strategies.

Beyond market fluctuations, our model did not account for the training and behavioral changes needed so that farm staff learn new procedures and improve management overall. Farmers’ attitude toward AMU including their knowledge of AMR, capabilities, social pressure and motivation play a role in adopting AMU reducing options (83). Regan et al. (84) argued that a bottom-up behavior change from an individual farmer to organizational and societal level interventions alongside implementation of regulatory policies are required for a wider adaptation of AMU reducing practices. We acknowledge that analysis of the uptake of AMU options based solely on farm gross margin is limiting. However, economic feasibility is always one of the main concerns of farmers and plays a major role in making decisions to uptake new farm practices (85). It is clear from the literature consulted and expert input that just reducing AMU (without any other measures) is not going to increase margins and could have a negative impact on animal welfare. Each farm must consider what is the most feasible and suitable set of improvements to accompany AMU reduction and should see this endeavor as a holistic attempt to improve health and welfare on farm. The approach chosen (i.e., improvements and management changes to be implemented) should be a joint decision between farmers, their staff, and the farm veterinarian.

## Data availability statement

The data analyzed in this study is subject to the following licenses/restrictions: The data used in the model is taken from the e-MB owned by AHDB, UK and is restricted to protect confidentiality of the UK pig farmers. Requests to access these datasets should be directed to https://emb-pigs.ahdb.org.uk/.

## Ethics statement

The animal study was approved by the Veterinary Medicine Directorate. The study was conducted in accordance with the local legislation and institutional requirements.

## Author contributions

SS: Writing – review & editing, Writing – original draft, Validation, Methodology, Investigation, Funding acquisition, Formal analysis, Conceptualization. MC: Writing – review & editing, Validation, Methodology, Formal analysis. CC-G: Writing – review & editing, Methodology, Funding acquisition, Conceptualization. AN: Writing – review & editing, Validation, Data curation. AM: Writing – review & editing, Validation, Data curation. ST: Writing – review & editing, Validation, Supervision, Resources.

## Footnotes

^1^Enzymes produced by *E. coli* – Extended spectrum β-lactamase (ESBL) and Ampicillin β-lactamase (AmpC).
